# 4-(3-Nitro­phen­yl)-3-(phenyl­sulfon­yl)but-3-en-2-one

**DOI:** 10.1107/S1600536808005370

**Published:** 2008-02-29

**Authors:** Yongjiang Wang, Wen Pei

**Affiliations:** a College of Biological and Chemical Engineering, Zhejiang University of Science and Technology, Hangzhou 310023, People’s Republic of China; b College of Chemical Engineering and Materials Science, Zhejiang University of Technology, Hangzhou 310014, People’s Republic of China

## Abstract

The C=C double bond in the title mol­ecule, C_16_H_13_NO_5_S, has an *E* configuration. The crystal structure is stabilized by C—H⋯O hydrogen bonds. There is also a weak C—H⋯π-ring inter­action in the structure.

## Related literature

For related literature, see: Pei (1998 ▶). For the chemical preparation, see: Wada *et al.* (1996 ▶).
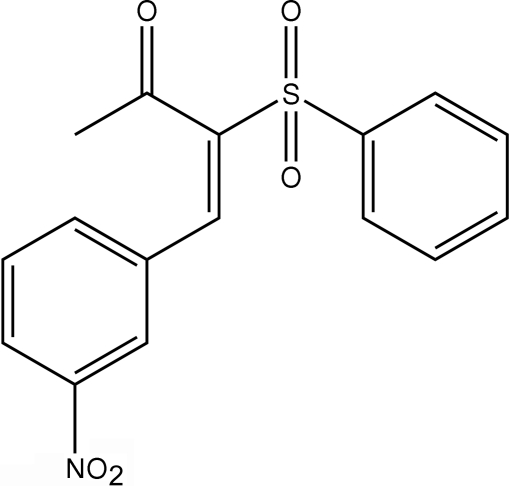


## Experimental

### 

#### Crystal data

C_16_H_13_NO_5_S
*M* *_r_* = 331.34
Monoclinic, 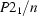

*a* = 7.957 (2) Å
*b* = 10.580 (3) Å
*c* = 18.271 (6) Å
β = 95.976 (14)°
*V* = 1529.7 (8) Å^3^
*Z* = 4
Mo *K*α radiation
μ = 0.24 mm^−1^
*T* = 298 (1) K
0.25 × 0.20 × 0.20 mm

#### Data collection

Rigaku R-AXIS RAPID diffractometer
Absorption correction: multi-scan (*ABSCOR*; Higashi, 1995 ▶) *T*_min_ = 0.923, *T*_max_ = 0.954
14836 measured reflections
3507 independent reflections
2890 reflections with *I* > 2σ(*I*)
*R*_int_ = 0.027

#### Refinement

*R*[*F* ^2^ > 2σ(*F* ^2^)] = 0.038
*wR*(*F* ^2^) = 0.106
*S* = 1.05
3507 reflections
210 parameters
H-atom parameters constrained
Δρ_max_ = 0.34 e Å^−3^
Δρ_min_ = −0.40 e Å^−3^

### 

Data collection: *PROCESS-AUTO* (Rigaku, 1998 ▶); cell refinement: *PROCESS-AUTO*; data reduction: *CrystalStructure* (Rigaku/MSC, 2004 ▶); program(s) used to solve structure: *SIR97* (Altomare *et al.*, 1999 ▶); program(s) used to refine structure: *SHELXL97* (Sheldrick, 2008 ▶); molecular graphics: *ORTEP-3 for Windows* (Farrugia, 1997 ▶); software used to prepare material for publication: *CrystalStructure*.

## Supplementary Material

Crystal structure: contains datablocks global, I. DOI: 10.1107/S1600536808005370/fb2081sup1.cif

Structure factors: contains datablocks I. DOI: 10.1107/S1600536808005370/fb2081Isup2.hkl

Additional supplementary materials:  crystallographic information; 3D view; checkCIF report

## Figures and Tables

**Table 1:** Hydrogen-bond geometry (Å, °)

*D*—H⋯*A*	*D*—H	H⋯*A*	*D*⋯*A*	*D*—H⋯*A*
C4—H4⋯O4^i^	0.93	2.60	3.104 (2)	114
C8—H8⋯O5^ii^	0.93	2.53	3.321 (2)	143
C10—H10⋯O3^iii^	0.93	2.54	3.364 (2)	148
C13—H13⋯O5^iii^	0.93	2.59	3.433 (2)	152
C12—H12⋯O4	0.93	2.52	2.903 (2)	105
C16—H16⋯O4^i^	0.93	2.60	3.524 (2)	176
C9—H9⋯*Cg*1^ii^	0.93	2.80	3.608 (2)	145

Symmetry codes: (i) 
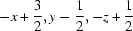
; (ii) 
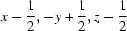
; (iii) 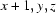. *Cg*1 is the centroid of the C11–C16 phenyl ring.

**Fig. 1.**
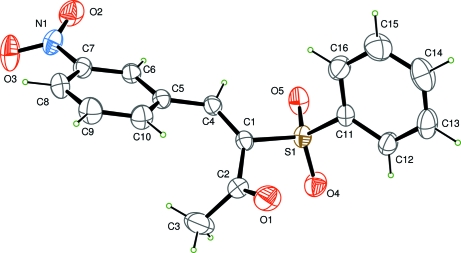
View of the title molecule showing the atom labelling scheme. The displacement ellipsoids are drawn at the 40% probability level.

## supplementary crystallographic information

### Comment

Phenylsulfonyl-3-alken-2-ones have been used as asymmetric synthetic reagents (Wada *et al.*, 1996) as well as electron-deficient dienophiles. They have been applied in asymmetric Diels–Alder chemistry (Pei, 1998). The title compound has been prepared and studied in order to get a better understanding about the synthesis of phenylsulfonyl-3-alken-2-ones. The molecular structure, Fig. 1, shows that an *E*-configuration is present on the C=C double bond between the atoms C1 and C4. There is also present a C—H···π-electron ring interaction in the structure. It involves the phenyl ring C11–C16 with centroid *Cg*1 (Table 1).

### Experimental

The title compound was prepared according to the procedure of Wada *et al.* (1996). Diffraction quality crystals were obtained by recrystallization from ethanol solution by slow evaporation at room temperature. The average dimension of the block crystals was about 0.2 mm.

### Refinement

All the H atoms were discerned in the difference Fourier map. Nevertheless, the H atoms were positioned into the idealized positions and their parameters were constrained in riding-mode approximation. The constraints: *C*—H_aryl_,_alkenyl_)=0.93 and *C*—H_methyl_=0.96 Å. *U*_iso_(H_aryl_,H_alkenyl_)= 1.2*U*_eq_(C_aryl_,*C*_alkenyl_) and *U*_iso_(H_metyl_)=1.5*U*_eq_(C_methyl_).

### Crystal data

**Table:**

C_16_H_13_NO_5_S	*F*_000_ = 688.00
*M**_r_* = 331.34	*D*_x_ = 1.439 Mg m^−^^3^
Monoclinic, *P*2_1_/*n*	Mo *K*α radiation λ = 0.71075 Å
Hall symbol: -P 2yn	Cell parameters from 12472 reflections
*a* = 7.957 (2) Å	θ = 3.2–27.5º
*b* = 10.580 (3) Å	µ = 0.24 mm^−^^1^
*c* = 18.271 (6) Å	*T* = 298 (1) K
β = 95.976 (14)º	Block, colourless
*V* = 1529.7 (8) Å^3^	0.25 × 0.20 × 0.20 mm
*Z* = 4	

### Data collection

**Table:**

Rigaku R-AXIS RAPID diffractometer	3507 independent reflections
Monochromator: graphite	2890 reflections with *I* > 2σ(*I*)
Detector resolution: 10.00 pixels mm^-1^	*R*_int_ = 0.027
*T* = 285 K	θ_max_ = 27.5º
ω scans	θ_min_ = 3.2º
Absorption correction: multi-scan(ABSCOR; Higashi, 1995)	*h* = −8→10
*T*_min_ = 0.923, *T*_max_ = 0.954	*k* = −13→13
14836 measured reflections	*l* = −23→23

### Refinement

**Table:**

Refinement on *F*^2^	Secondary atom site location: difference Fourier map
Least-squares matrix: full	Hydrogen site location: difference Fourier map
*R*[*F*^2^ > 2σ(*F*^2^)] = 0.038	H-atom parameters constrained
*wR*(*F*^2^) = 0.106	*w* = 1/[σ^2^(*F*_o_^2^) + (0.0516*P*)^2^ + 0.4387*P*] where *P* = (*F*_o_^2^ + 2*F*_c_^2^)/3
*S* = 1.05	(Δ/σ)_max_ = 0.001
3507 reflections	Δρ_max_ = 0.34 e Å^−^^3^
210 parameters	Δρ_min_ = −0.40 e Å^−^^3^
42 constraints	Extinction correction: SHELXL97 (Sheldrick, 2008)
Primary atom site location: structure-invariant direct methods	Extinction coefficient: 0.0097 (17)

### Special details

**Table:**

Geometry. ENTER SPECIAL DETAILS OF THE MOLECULAR GEOMETRY
Refinement. Refinement using all reflections. The weighted *R*-factor (*wR*) and goodness of fit (*S*) are based on *F*^2^. *R*-factor (gt) are based on *F*. The threshold expression of *F*^2^ > 2.0 σ(*F*^2^) is used only for calculating *R*-factor (gt).

### Fractional atomic coordinates and isotropic or equivalent isotropic displacement parameters (Å^2^)

**Table:**

	*x*	*y*	*z*	*U*_iso_*/*U*_eq_	
S1	0.84122 (5)	0.43647 (4)	0.25330 (2)	0.03894 (14)	
O1	0.99494 (16)	0.40871 (14)	0.10399 (8)	0.0607 (4)	
O2	0.02708 (17)	0.12269 (16)	0.11304 (9)	0.0674 (4)	
O3	−0.02286 (17)	0.14516 (19)	−0.00423 (9)	0.0806 (5)	
O4	0.89564 (17)	0.56378 (11)	0.24210 (8)	0.0568 (4)	
O5	0.72060 (15)	0.41361 (14)	0.30472 (7)	0.0566 (4)	
N1	0.07214 (18)	0.14380 (15)	0.05295 (9)	0.0508 (4)	
C1	0.75310 (18)	0.37984 (14)	0.16622 (8)	0.0349 (3)	
C2	0.8455 (2)	0.42725 (15)	0.10363 (9)	0.0418 (4)	
C3	0.7499 (3)	0.5076 (2)	0.04658 (14)	0.0770 (7)	
H31	0.8105	0.5121	0.0039	0.116*	
H32	0.7371	0.5910	0.0659	0.116*	
H33	0.6404	0.4713	0.0332	0.116*	
C4	0.61399 (18)	0.30895 (15)	0.16516 (8)	0.0370 (3)	
H4	0.5739	0.2956	0.2106	0.044*	
C5	0.51608 (18)	0.24949 (14)	0.10202 (8)	0.0357 (3)	
C6	0.34611 (19)	0.22278 (15)	0.10721 (9)	0.0376 (3)	
H6	0.2974	0.2406	0.1502	0.045*	
C7	0.25178 (19)	0.16961 (15)	0.04761 (9)	0.0385 (3)	
C8	0.3182 (2)	0.13919 (17)	−0.01672 (9)	0.0473 (4)	
H8	0.2507	0.1052	−0.0565	0.057*	
C9	0.4884 (2)	0.16088 (19)	−0.02025 (10)	0.0526 (4)	
H9	0.5374	0.1386	−0.0625	0.063*	
C10	0.5862 (2)	0.21504 (17)	0.03795 (10)	0.0462 (4)	
H10	0.7005	0.2289	0.0346	0.055*	
C11	1.02028 (18)	0.34291 (15)	0.28003 (8)	0.0369 (3)	
C12	1.1754 (2)	0.40079 (18)	0.29545 (9)	0.0453 (4)	
H12	1.1883	0.4867	0.2868	0.054*	
C13	1.3113 (2)	0.3282 (2)	0.32406 (11)	0.0590 (5)	
H13	1.4168	0.3654	0.3350	0.071*	
C14	1.2909 (3)	0.2017 (2)	0.33632 (12)	0.0655 (6)	
H14	1.3824	0.1538	0.3563	0.079*	
C15	1.1369 (3)	0.1452 (2)	0.31942 (13)	0.0658 (6)	
H15	1.1252	0.0589	0.3271	0.079*	
C16	0.9995 (2)	0.21509 (17)	0.29120 (11)	0.0524 (4)	
H16	0.8947	0.1770	0.2799	0.063*	

### Atomic displacement parameters (Å^2^)

**Table:**

	*U*^11^	*U*^22^	*U*^33^	*U*^12^	*U*^13^	*U*^23^
S1	0.0325 (2)	0.0432 (2)	0.0403 (2)	0.00241 (15)	−0.00025 (15)	−0.00634 (16)
O1	0.0424 (7)	0.0742 (9)	0.0676 (9)	−0.0004 (6)	0.0163 (6)	0.0104 (7)
O2	0.0468 (7)	0.0850 (11)	0.0725 (10)	−0.0159 (7)	0.0157 (7)	−0.0016 (8)
O3	0.0393 (7)	0.1266 (15)	0.0713 (10)	0.0022 (8)	−0.0154 (7)	−0.0245 (10)
O4	0.0583 (8)	0.0375 (6)	0.0717 (9)	0.0017 (5)	−0.0075 (7)	−0.0083 (6)
O5	0.0395 (6)	0.0887 (10)	0.0420 (7)	0.0056 (6)	0.0062 (5)	−0.0108 (6)
N1	0.0348 (7)	0.0560 (9)	0.0606 (10)	−0.0022 (6)	0.0003 (7)	−0.0121 (7)
C1	0.0321 (7)	0.0371 (7)	0.0346 (7)	0.0026 (6)	−0.0009 (6)	0.0016 (6)
C2	0.0411 (8)	0.0418 (8)	0.0423 (9)	−0.0043 (7)	0.0038 (7)	0.0017 (6)
C3	0.0709 (14)	0.0856 (16)	0.0746 (15)	0.0032 (12)	0.0080 (12)	0.0424 (13)
C4	0.0335 (7)	0.0425 (8)	0.0344 (7)	0.0013 (6)	0.0012 (6)	0.0035 (6)
C5	0.0325 (7)	0.0384 (7)	0.0355 (7)	0.0008 (6)	0.0004 (6)	0.0036 (6)
C6	0.0345 (7)	0.0437 (8)	0.0348 (8)	−0.0007 (6)	0.0047 (6)	0.0001 (6)
C7	0.0319 (7)	0.0412 (8)	0.0413 (8)	0.0020 (6)	−0.0006 (6)	0.0001 (6)
C8	0.0476 (9)	0.0549 (10)	0.0377 (8)	0.0027 (8)	−0.0039 (7)	−0.0071 (7)
C9	0.0516 (10)	0.0671 (12)	0.0406 (9)	0.0026 (9)	0.0128 (8)	−0.0096 (8)
C10	0.0356 (8)	0.0568 (10)	0.0472 (9)	0.0010 (7)	0.0086 (7)	−0.0031 (8)
C11	0.0317 (7)	0.0447 (8)	0.0336 (7)	0.0004 (6)	0.0004 (6)	−0.0030 (6)
C12	0.0347 (8)	0.0575 (10)	0.0435 (9)	−0.0072 (7)	0.0031 (7)	−0.0021 (7)
C13	0.0308 (8)	0.0930 (16)	0.0526 (11)	0.0023 (9)	0.0016 (7)	−0.0053 (10)
C14	0.0525 (11)	0.0838 (15)	0.0587 (12)	0.0304 (11)	−0.0011 (9)	−0.0044 (11)
C15	0.0724 (14)	0.0492 (11)	0.0744 (14)	0.0161 (10)	0.0006 (11)	−0.0012 (9)
C16	0.0471 (10)	0.0451 (9)	0.0633 (11)	−0.0017 (7)	−0.0020 (8)	−0.0047 (8)

### Geometric parameters (Å, °)

**Table:**

S1—O5	1.4317 (13)	C6—H6	0.9300
S1—O4	1.4360 (13)	C7—C8	1.376 (2)
S1—C11	1.7617 (16)	C8—C9	1.382 (3)
S1—C1	1.7747 (16)	C8—H8	0.9300
O1—C2	1.204 (2)	C9—C10	1.375 (2)
O2—N1	1.211 (2)	C9—H9	0.9300
O3—N1	1.224 (2)	C10—H10	0.9300
N1—C7	1.468 (2)	C11—C16	1.380 (2)
C1—C4	1.335 (2)	C11—C12	1.381 (2)
C1—C2	1.508 (2)	C12—C13	1.384 (3)
C2—C3	1.491 (3)	C12—H12	0.9300
C3—H31	0.9600	C13—C14	1.369 (3)
C3—H32	0.9600	C13—H13	0.9300
C3—H33	0.9600	C14—C15	1.369 (3)
C4—C5	1.465 (2)	C14—H14	0.9300
C4—H4	0.9300	C15—C16	1.375 (3)
C5—C6	1.394 (2)	C15—H15	0.9300
C5—C10	1.396 (2)	C16—H16	0.9300
C6—C7	1.377 (2)		
			
O5—S1—O4	118.99 (9)	C8—C7—C6	123.02 (15)
O5—S1—C11	107.53 (8)	C8—C7—N1	118.44 (14)
O4—S1—C11	108.63 (8)	C6—C7—N1	118.54 (14)
O5—S1—C1	107.57 (8)	C7—C8—C9	117.81 (15)
O4—S1—C1	106.61 (8)	C7—C8—H8	121.1
C11—S1—C1	106.95 (7)	C9—C8—H8	121.1
O2—N1—O3	124.14 (16)	C10—C9—C8	120.74 (16)
O2—N1—C7	118.48 (15)	C10—C9—H9	119.6
O3—N1—C7	117.38 (16)	C8—C9—H9	119.6
C4—C1—C2	130.13 (14)	C9—C10—C5	120.92 (15)
C4—C1—S1	116.88 (12)	C9—C10—H10	119.5
C2—C1—S1	112.89 (11)	C5—C10—H10	119.5
O1—C2—C3	121.90 (17)	C16—C11—C12	121.53 (15)
O1—C2—C1	120.05 (15)	C16—C11—S1	119.05 (12)
C3—C2—C1	117.76 (16)	C12—C11—S1	119.18 (13)
C2—C3—H31	109.5	C11—C12—C13	118.54 (18)
C2—C3—H32	109.5	C11—C12—H12	120.7
H31—C3—H32	109.5	C13—C12—H12	120.7
C2—C3—H33	109.5	C14—C13—C12	120.20 (18)
H31—C3—H33	109.5	C14—C13—H13	119.9
H32—C3—H33	109.5	C12—C13—H13	119.9
C1—C4—C5	128.65 (14)	C15—C14—C13	120.55 (18)
C1—C4—H4	115.7	C15—C14—H14	119.7
C5—C4—H4	115.7	C13—C14—H14	119.7
C6—C5—C10	118.61 (14)	C14—C15—C16	120.5 (2)
C6—C5—C4	118.26 (14)	C14—C15—H15	119.7
C10—C5—C4	123.10 (14)	C16—C15—H15	119.7
C7—C6—C5	118.80 (14)	C15—C16—C11	118.64 (18)
C7—C6—H6	120.6	C15—C16—H16	120.7
C5—C6—H6	120.6	C11—C16—H16	120.7
			
O4—S1—C1—C2	36.47 (12)	C4—C1—C2—C3	58.5 (2)
O4—S1—C1—C4	−140.16 (11)	C1—C4—C5—C6	−155.46 (15)
O4—S1—C11—C12	8.06 (15)	C1—C4—C5—C10	26.5 (2)
O4—S1—C11—C16	−177.55 (14)	C4—C5—C6—C7	178.54 (13)
O5—S1—C1—C2	165.12 (10)	C4—C5—C10—C9	−179.12 (15)
O5—S1—C1—C4	−11.50 (13)	C6—C5—C10—C9	2.9 (2)
O5—S1—C11—C12	−121.99 (13)	C10—C5—C6—C7	−3.4 (2)
O5—S1—C11—C16	52.59 (15)	C5—C6—C7—N1	−178.84 (13)
C1—S1—C11—C12	122.77 (13)	C5—C6—C7—C8	1.3 (2)
C1—S1—C11—C16	−62.81 (15)	N1—C7—C8—C9	−178.34 (14)
C11—S1—C1—C2	−79.59 (11)	C6—C7—C8—C9	1.6 (2)
C11—S1—C1—C4	103.79 (12)	C7—C8—C9—C10	−2.2 (2)
O2—N1—C7—C6	−28.2 (2)	C8—C9—C10—C5	−0.0 (2)
O2—N1—C7—C8	151.86 (15)	S1—C11—C12—C13	173.00 (13)
O3—N1—C7—C6	151.83 (16)	S1—C11—C16—C15	−173.37 (15)
O3—N1—C7—C8	−28.2 (2)	C12—C11—C16—C15	1.0 (2)
S1—C1—C2—O1	56.37 (18)	C16—C11—C12—C13	−1.3 (2)
S1—C1—C2—C3	−117.54 (15)	C11—C12—C13—C14	0.2 (2)
S1—C1—C4—C5	−179.53 (12)	C12—C13—C14—C15	1.0 (3)
C2—C1—C4—C5	4.5 (2)	C13—C14—C15—C16	−1.3 (3)
C4—C1—C2—O1	−127.56 (18)	C14—C15—C16—C11	0.3 (3)

### Hydrogen-bond geometry (Å, °)

**Table:**

*D*—H···*A*	*D*—H	H···*A*	*D*···*A*	*D*—H···*A*
C4—H4···O4^i^	0.93	2.60	3.104 (2)	114
C8—H8···O5^ii^	0.93	2.53	3.321 (2)	143
C10—H10···O3^iii^	0.93	2.54	3.364 (2)	148
C13—H13···O5^iii^	0.93	2.59	3.433 (2)	152
C12—H12···O4	0.93	2.52	2.903 (2)	105
C16—H16···O4^i^	0.93	2.60	3.524 (2)	176
C9—H9···Cg1^ii^	0.93	2.80	3.608 (2)	145

Symmetry codes: (i) −*x*+3/2, *y*−1/2, −*z*+1/2; (ii) *x*−1/2, −*y*+1/2, *z*−1/2; (iii) *x*+1, *y*, *z*.
